# Evaluation of Dietary Curcumin Nanospheres in a Weaned Piglet Model

**DOI:** 10.3390/antibiotics10111280

**Published:** 2021-10-20

**Authors:** Mohammad Moniruzzaman, Hunhwan Kim, Haewon Shin, Hyunsoo Kim, Nayoung Kim, Sungyeon Chin, Adhimoolam Karthikeyan, Hyojick Choi, Gonsup Kim, Taesun Min

**Affiliations:** 1 Department of Animal Biotechnology, Jeju International Animal Research Center (JIA) & Sustainable Agriculture Research Institute (SARI), Jeju National University, Jeju 63243, Korea; monir1983@jejunu.ac.kr (M.M.); jjkanji23@naver.com (H.S.); rlagustn9891@naver.com (H.K.); skdud559@naver.com (N.K.); syeun77777@naver.com (S.C.); 2 Research Institute of Life Science and College of Veterinary Medicine, Gyeongsang National University, Gazwa, Jinju 52828, Korea; shark159753@naver.com; 3 Subtropical Horticulture Research Institute, Jeju National University, Jeju 63243, Korea; karthik2373@gmail.com; 4 Sustainable Engineering & Drug Delivery Design Lab, Department of Chemical and Materials Engineering, University of Alberta, Edmonton, AB T6G 2V4, Canada; hyojick@ualberta.ca

**Keywords:** nanomedicine, alternative of antibiotics, growth, hematology, proteomics, coliform bacteria, noxious gas, piglet

## Abstract

Curcumin is a polyphenolic compound present in turmeric with extensive uses in cooking foods and biomedical applications. However, due to its hydrophobic nature, it is poorly soluble in water and its bioavailability is very low on oral administration in organisms. In this study, we investigated the dietary curcumin nanospheres in a weaned piglet model based on the growth, serum biochemistry, proteomics, fecal coliform bacteria, and malodors in the feces of piglets. A total of 135 weaned piglets (Duroc × [Yorkshire × Landrace]) with an average initial body weight of 7.0 ± 1.0 kg (28 ± 1 days of age) were randomly distributed in 9 pens (15 pigs in each pen) fed the dietary curcumin nanospheres (CN) at 0 (control), 0.5 (T1), and 1.0 mL (T2) CN/kg of diet in triplicates for 21 days. At the end of the feeding trial, the results showed piglets fed 1.0 mL CN/kg diet had significantly higher growth performance and feed utilization than control diet (without CN). However, there were no significant differences in growth and feed utilization between piglets fed T1 and T2 diets. Serum glucose, gamma-glutamyl transferase, total bilirubin, amylase, and lipase contents were unaffected in piglets fed the experimental diets. Interestingly, piglets fed T1 and T2 diets showed significantly lower total cholesterol levels than control diet. In serum proteomics, a total of 103 differentially expressed proteins (DEPs) were identified in the piglets fed control, T1, and T2 diets, of which 14 DEPs were upregulated and 4 DEPs were downregulated. Fecal coliform bacteria and ammonia gas were significantly reduced in piglets fed T1 and T2 diets. Overall, the results indicated dietary supplementation of CN could enhance the growth, feed utilization, and immunity—and reduce fecal pathogenic bacteria as well as ammonia gas emissions—in weaned piglets.

## 1. Introduction

Curcumin is a lipophilic polyphenolic phytocompound present in the herbal plant, *Curcuma longa* which has been widely used as turmeric powder in Southeast Asian countries [1]. Curcumin has been reported to act as a potential therapeutic agent due to its antioxidant, anti-inflammatory, antimicrobial, and anticancer activities [2,3,4]. For its therapeutic significance, curcumin would treat or prevent different types of ailments such as arthritis, enteritis, stomach ulcers, wounds, acne, skin lesions, neurodegeneration, and cancers [5]. The turmeric plant is known to contains nearly about 3–6% of highly bioactive curcumin with low toxicity in the form of diferuloylmethane [1,7-bis(4-hydroxy-3-methoxyphenyl)-1,6-heptadiene-3,5-dione] in a dry condition and generally regarded as safe (GRAS) by United States Food Drug Administration (USFDA) [6,7]. For centuries, curcumin has been widely used as Ayurvedic herbal medicine in India and China [5]. However, curcumin has low solubility in water, rapid excretion, but poor absorption and bioavailability in organisms, making its utilization challenging [8]. About 75% of curcumin can be excreted from feces after its oral administration in rats [9]. For this, a significant number of studies have been conducted to increase the solubility and bioavailability of curcumin in the form of nanoparticles or nanocurcumin in relation to the normal curcumin [10,11,12,13,14]. Nanopharmaceutics or nanomedicines are usually developed on the nanosize and its physicochemical characteristics, pharmacokinetics as well as biological activity at molecular level [15]. In this regard, nanocurcumin which coined as particle sizes of curcumin with less than 1 μm, has a great importance as nanomedicine in pharmaceutical and biomedical science [16]. However, the nanosizing of curcumin and its effective delivery in organisms is the prime concern in suggesting the potentiality of nanocurmin. In this case, the use of carrier vesicles or nanocarriers could offer a promising approach in terms of effective biodistribution of nanocurcumin as an active pharmaceutical ingredient (API) in organisms [15]. Specifically, curcumin can be encapsulated in nanocarriers such as nanoemulsions, nanoliposomes, solid lipid nanoparticles (SLNs), or polymer-based techniques (i.e., PLGA nanoparticles, biopolymers, hydrogels, or nature-inspired techniques like casein and cyclodextrins) [7,17,18,19,20,21,22]. According to a report, PLGA-nanocurcumin at 15-fold lower concentration is superior to poorly soluble native curcumin in therapeutic application [23], while another study suggested that encapsulation of nanocurcumin is 9-times more suitable than native curcumin in terms of oral bioavailability in animals [24]. Verma et al. [25] concluded that liposomal nanocarriers are highly biocompatible and can entrap hydrophobic pharmaceuticals in the membrane. In other studies, hydrophobic nanocurcumin with liposomal nanocarriers are found to be highly effective in biomedical applications [26,27,28]. Liposomal nanoparticles consisting in phosphatidylcholine or phosphatidylserine and containing curcumin exerted their potential in terms of oral delivery for bioavailability, antioxidant properties, and treatment of hepatic fibrosis [26,29].

Numerous studies have been conducted to investigate the biomedical applications of nanocurcumin with different nanocarriers using in vitro cell lines or in vivo murine models [30]. However, little is known about the utilization of nanocurcumin in large animal models [31,32]. Importantly, swine has been discussed as a promising monogastric large animal in investigating digestive physiology, immunology, and toxicology concerning human diseases [33,34,35]. According to a study, a pig’s immune system is about 80% similar to that of a human being in comparison to a mouse—which resembles only about 10%— making pigs an excellent model for testing the effect of nanocurcumin in humans along with the fact that pigs can be easily bred compared to other large animals like monkeys and dogs [34]. In the life cycle of pig, young pigs experience diarrheal problems, complications in the gastrointestinal tract (GIT), and stunted growth with high morbidity and mortality during their weaning [36]. In addition, pig farms are a major source of noxious gases such as ammonia (NH_3_) and hydrogen sulfide (H_2_S), which affect the air quality of the surrounding areas including human livelihoods. Therefore, it is of the utmost importance to determine a suitable way to enhance the growth and immunity—and reduce the diarrheal problem—in young piglets and reduce fecal malodors from the pig farms. In this regard, we consider curcumin nanospheres (CN) a potent treatment for them, as Liu et al. [37] and Kim et al. [38] postulated that dietary plant extracts have significant positive effects on immune responses in their proteomics analyses of weaned pigs. 

In the recent studies of our research group, we have reported the positive effects of curcumin nanospheres (CN) on using in vitro cell lines and in vivo mouse model [39,40] including the physicochemical characteristics and pharmacokinetics in terms of size, solubility, efficiency, drug delivery, and bioavailability of CN [41]. In the present study, we envisage to evaluate the dietary effects of curcumin nanospheres with pharmacodynamics in weaned piglets as a large monogastric animal model based on growth performance, serum biochemistry, serum proteomic analysis, fecal coliform bacteria contents and fecal malodors in feces.

## 2. Materials and Methods

### 2.1. Ethics Statement

The experiment was followed under the guidelines of Institutional Animal Care and Use Committee of the Jeju National University, Jeju, Republic of Korea (approval number: 2021-0055). Every effort was taken to minimize animal suffering.

### 2.2. Chemicals and Reagents

Curcumin powder (from *Curcuma longa*, turmeric plant) and lecithin (in the form of l-α-phosphatidylcholine from egg yolk) were purchased from Sigma-Aldrich (St. Louis, MO, USA). The organic solvents, toluene (anhydrous, 99.8%) and dichloromethane (DCM, 99.9%), were obtained from Sigma-Aldrich (St. Louis, MO, USA) and Acros Organics (Janssen Pharmaceuticals, Geel, Belgium), respectively. All other chemicals and reagents were analytical grade.

### 2.3. Preparation of Curcumin Nanospheres (CN)

Curcumin nanospheres were produced as described in previous reports [39,40,41]. Briefly, 100 mg curcumin powder was dissolved in 20 mL toluene as organic solvent and then the solution was added dropwise in 1 L distilled water on heating mantle with continuous ultrasonication at a frequency of 50 kHz for 3 h. The sonication process was operated within an insulated sonication box with sonicating probe (Sonictopia, Rep. of Korea) to reduce hazardous effects of ultrasonication on the outside environment. After sonication, the solution was stirred for 20 min on the heating mantle with a magnetic stirrer. The solution was then transferred to a rotary evaporator (Buchi AG, Meierseggstrasse, Flawil, Switzerland) and concentrated the solution at the evaporating temperature of 40 °C. The concentrated solution was freeze dried for 72 h to obtain nanocurcumin powder. For the production of curcumin nanospheres, 200 mg of lecithin was dissolved in 40 mL dichloromethane (DCM) and then 40 mg of the nanocurcumin powder was mixed with the lecithin-DCM solution (1 mg of nanocurcumin powder with 5 mg lecithin/ mL DCM). The mixture was ultrasonicated for 2 h at 20–30 kHz to achieve an orange-colored curcumin nanospheres (CN) solution. The CN solution was stored in falcon tubes at −70 °C until used in the feeds of weaned piglets. The CN was produced at large scale to execute the in vivo experiment in weaned piglets. Furthermore, morphology and characteristics of CN was described by Kim et al. [39].

### 2.4. Addition of CN in the Diets of Piglets

A commercial powder diet (Neopigg, Purina Korea) contained with all the nutritional requirements (protein, 23.0%; lipid, 5.5%; ash, 8.0%; fiber, 4.0%; digestible energy, 3.6 Mcal/kg) for weaned piglets was used as the control without supplementation of CN. Two other diets were prepared with dropwise mixing of CN at 0.5 mL/kg (T1) and 1.0 mL/kg (T2) in the powder of control diet using a mixer based on Marcon et al. [31]. The diets were then dried at 25 °C room temperature (RT) for 72 h to remove the DCM in the diets. The three diets were then packed in airtight bags labeled with the diet numbers.

### 2.5. Animals and Experimental Design

A total of 135 purchased weaned piglets (Duroc × [Yorkshire × Landrace]) with an average initial body weight of 7.0 ± 1.0 kg and 28 ± 1 days of age were used in this study. The experiment was executed in a local pig farm (Bada Pig Farm, Hallim, Jeju, Republic of Korea. The piglets were randomly distributed into 3 groups in triplicates (15 piglets/pen and 45 piglets/treatments) according to the designated diets of the control, T1, and T2. All the piglets were marked by numbers on the skin with permanent red ink marker for easy identification during sampling and the pens were equipped with drinking water, controlled temperature, feed supply, and drainage facilities. The piglets were fed the experimental feeds and water ad libitum. The temperature in the rearing pens was maintained at 28.0 ± 1.0 °C throughout the experimental period.

### 2.6. Sample Collection and Analyses of Piglets

#### 2.6.1. Growth Parameters

At the end of the 21-day feeding trial, the weights of the piglets were measured individually in weight cages according to the pen number and treatment group numbers following the weight gain (WG), average daily gain (ADG), feed intake (FI), average feed intake (ADFI), feed efficiency (FE), and feed conversion ratio (FCR) were calculated based on initial weight, final weight (FW), and daily feed intake data. Fresh fecal samples were obtained from each pen of the treatment groups based on the dietary treatments.

#### 2.6.2. Serum Biochemical Composition

For examining the serum biochemistry, blood samples from the jugular vein of 3 pigs (based on average weight) in each pen of the three treatment groups were collected for biochemical analyses. All of the samples were allowed to rest for few minutes at room temperature (RT), followed by centrifugation 1500× *g* at 4 °C for 20 min [42]. The supernatant of each blood sample (blood serum) was collected by micropipette and kept in a microtube at −20 °C until analyzed. The serum biochemistry in terms of glucose (Glu), creatinine (CRT), blood urea nitrogen (BUN), inorganic phosphorus (IP), calcium (Ca), total protein (T-Pro), albumin (ALB), globulin (GLB), alanine aminotransferase (ALT), alkaline phosphatase (ALKP), gamma-glutamyl transferase (GGT), total bilirubin (TBIL), total cholesterol (TCHOL), amylase (AMYL), and lipase (LYPS) was determined by commercial kits using a blood biochemical analyzer (Refloton Plus, Hoffmann-La Roche, Rotkreuz, Switzerland).

#### 2.6.3. Serum Proteome Analysis

##### Sample Preparation

For serum proteomic analysis, an aliquot (5 mL) of collected blood serum was analyzed based on the dietary treatments in the weaned piglets. For this, the serum samples were subjected to a gene ontology (GO) study followed by Kim et al. [43]. The protein concentrations in the serum samples were analyzed by using a bicinchoninic acid (BCA) assay kit (Pierce BCA protein assay kit, Thermo Fisher Scientific). Afterward, the samples with the same concentrations of protein were used for proteomic analysis.

##### Gel Electrophoresis

For one-dimensional SDS-PAGE, a total of 20 μg of proteins from the pig serum were diluted with a denaturing sample buffer (0.5 M Tris-HCl pH 8.8, 10% SDS, 20% glycerol, 1% bromophenol blue, 0.2% DTT), and heated at 95 °C for 5 min. Then the samples were transferred to a gel electrophoresis unit for SDS-PAGE. The gel was then stained with Coomassie Brilliant R250 (Sigma-Aldrich, ST. Louis, MO, USA) and de-stained with water [44]. All protein bands after the electrophoresis were excised using a razor blade from top to bottom, and the excised gel slices were washed twice with 100 µL of distilled water for 15 min at RT. The dried gel slices were then stored at −80 °C for further analysis.

##### Nano-LC-MS/MS Analysis

The dried peptides on the gel slices were dissolved in 20 µL of 5 % formic acid and analyzed using nanoflow LC-MS/MS. All nano-LC-MS/MS experiments were performed on an Ekisigent nanoLC415 system (EKsigent, Dublin, CA, USA). For a gel-free proteomic analysis, the protein samples were re-suspended in a water/formic acid solution (water in 5% formic acid). Online NanoHPLC was conducted using an Ekisigent nanoLC415 system (EKsigent, Dublin, CA, USA).

##### Data Search and Criteria for Protein Identification

After MS/MS analysis, the data files were processed using the UniProt and Protein Pilot 5.0.1 (SCIEX, Redwood City, CA, USA) database software. Based on the combined MS and MS/MS spectra, the proteins were successfully identified at a 95% or higher confidence interval, making it possible to use their scores in the MASCOT V2.5 search engine (Matrix Science Ltd., London, UK) and the following search parameters for *Sus scrofa* (Pig). The database search results were manually curated to yield the protein identifications using 1% global false discovery rate (FDR) determined by the in-built FDR tool within the ProteinPilot software and Scaffold (version Scaffold_4.8.4, Proteome Software Inc., Portland, OR, USA) was used to validate the MS/MS based peptide and protein identifications. The protein identification was based on 99% protein threshold and 95% peptide threshold levels. The differentially expressed proteins (DEPs) were categorized into upregulated and downregulated proteins present in the serum among piglets fed T1 and T2 diets in comparison to the control diet based on the log-fold change criteria, +1 (upregulated proteins) or −1 (downregulated proteins) cut off values.

##### Gene Ontology (GO) and Enrichment Pathway Analyses

The gene ontology (GO) categories of the serum protein of piglets fed the experimental diets were described based on biological processes, cellular components and molecular functions using Scaffold software. Likewise, all of the functionally enriched pathways of the significantly expressed proteins were determined with using the Kyoto Encyclopedia of Genes and Genomes (KEGG) and REACTOME pathway databases.

##### Protein–Protein Interaction (PPI) Network Analysis

The protein–protein interactions (PPI) using the Search Tool for the Retrieval of Interacting Genes (STRING, version 9.1) (http://string-db.org) (accessed on 16 September 2021) was used to determine both physical and functional associations between proteins. The PPI score was set based on medium confidence (0.4). In PPI network, each node depicts a protein, while the edges represent the strength of the relationship between proteins (i.e., more edges give higher confidence). The software Cytoscape was used to visualize the network.

#### 2.6.4. Fecal Noxious Gases and Pathogenic Bacteria Contents

The fecal samples collected from the same pen were mixed and stored in airtight plastic bags at −20 °C until the measurement of fecal malodors. Parts of the fresh fecal samples were placed in an icebox and pathogenic bacterial load analyses were immediately conducted in the laboratory. Following that, fecal samples in the bags were connected to a specific gas sampling kit for ammonia (NH_3_) and hydrogen sulfide (H_2_S) gases and then the fecal gases were measured in ppm (parts per million) by color changes method using a pumping apparatus (model GV-100S; Gastec Corp., Tokyo, Japan). Additionally, the pathogenic bacteria contents, such as *Escherichia coli* in the fecal samples were detected using the MacConkey agar plate counting method. Briefly, 1 g of a fecal sample from each pen was diluted and homogenized with 9 mL peptone water (15 g/L) (Oxoid Ltd., Hants, UK) and by vortexing, respectively. The homogenates were serially 10-fold diluted and spread (1 mL) on the MacConkey agar (51.5 g/L) plates (Oxoid Ltd., Hants, UK) followed by incubation at 37 °C for 24 h. After incubation, the coliform bacterial colonies were immediately counted using a colony counter and the data were presented in colony forming units per gram of sample (CFU/g).

### 2.7. Statistical Analysis

Piglets pen mean values (*n* = 3) were used for statistical analysis. All the data were subjected to a one-way analysis of variance (ANOVA) test using the SAS version 9.1 analytical software (SAS Institute, Cary, NC, USA) to test for the dietary treatments. Tukey’s HSD (honestly significant difference) *post-hoc* test was used to compare the means amongst the treatments with significant effects. Data values were expressed as mean ± standard deviation (SD) of the three replicates of each treatment group. Treatment effects were considered with a significant level of *p* < 0.05.

## 3. Results

### 3.1. Growth and Feed Intake of Piglets

The effects of dietary CN on the growth and feed intake of weaned piglets are presented in Table 1. At the end of the 21-day feeding trial, piglets fed with the T2 diet (1.0 mL CN/kg) showed significantly higher growth performance in terms of FW, WG, and ADG than those of the piglets fed control (C) diet (*p* < 0.05). However, there were no significant differences in growth performance of the piglets fed between the C and T1 (0.5 mL CN/kg) diet groups as well as between the T1 and T2 diet groups. Moreover, no significant difference was observed between FI and ADFI. However, the piglets fed T2 diet showed a significantly higher FE than the piglets fed C diet (*p* < 0.05). However, there were no significant differences in terms of FE between the groups of piglets fed C and T1 diets or between the T1 and T2 diets. Likewise, the FCR of piglets fed T2 diet showed a significantly lower value than those in C diet (*p* < 0.05). However, there were no significant difference in FCR between the groups of the piglets fed C and T1 diets or between T1 and T2 diets. No mortality of piglets was observed throughout the experimental period.

### 3.2. Serum Biochemistry of Piglets

The effects of dietary CN on the serum biochemistry of weaned piglets are presented in Table 2. As shown in the table, no significant differences in serum GLU, GGT, TBIL, AMYL, and LYPS contents were observed from the C, T1, and T2 diet groups. The piglets fed T2 diet showed significantly higher CRT contents than those in the C and T1 diets, but no significant difference was observed between C and T1 diets. Significantly higher BUN contents were found in the piglets fed T2 diet than in the C diet; however, there were no significant differences in BUN contents of the piglets fed C and T1 diets and between the T1 and T2 diets. Furthermore, there were no significant differences in BUN:CRT among the piglets fed three experimental diets. The piglets fed T2 diet had significantly higher IP and Ca contents than in the C and T1 diets. However, significantly lower amounts of IP and Ca contents were found in the piglets fed C diet than those in the T1 and T2 diets. Significantly higher T-Pro and ALKP contents were found in piglets fed T2 diet than those in the C and T1 diets, but no significant difference was found between the C and T1 diet groups. ALT level in piglets fed T2 diet had significantly higher than the piglets fed C and T1 diets. However, there were no significant differences in serum ALT level between the piglets fed C and T1 diets. The serum total cholesterol (TCHOL) content in the piglets fed C diet was significantly higher than those of the T1 and T2 diets, and a significantly lower TCHOL level was found in the piglets fed T2 diet than those in the C and T1 diets. The serum ALB content in the piglets fed T2 diet was significantly higher than the piglets fed C and T1 diets. There were no significant differences in serum GLB content among the piglets fed C, T1, and T2 diets. Furthermore, no significant difference in ALB:GLB was found in the piglets fed three experimental diets.

### 3.3. Serum Proteomics Analysis

#### 3.3.1. Identification of Differentially Expressed Proteins (DEPs)

**Figure 1:**
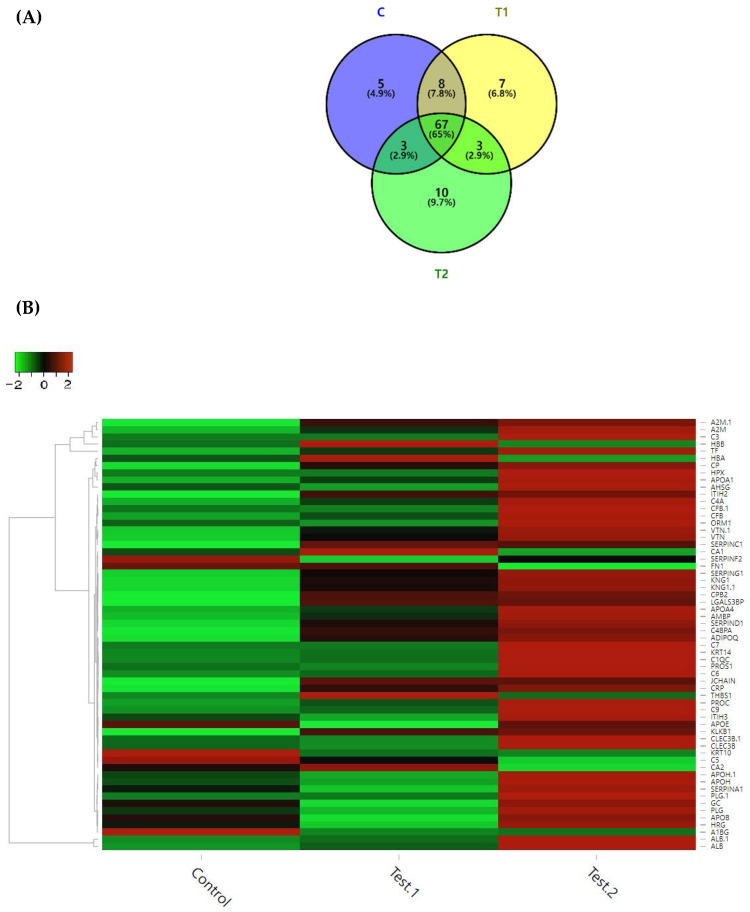
(**A**) Venn diagrams of the number and percentage of the identified differentially expressed protein (DEPs) and (**B**) Hierarchical clustering of proteins with significance level *p* < 0.05 for comparison of DEPs in the blood serum of the weaned piglets fed C (control), T1 (0.5 mL CN/kg), and T2 (1.0 mL CN/kg) diets. In heat map, the color scale illustrates the relative expression level of each protein across the samples; red and green colors indicate increased and decreased level of proteins, respectively, compared to the median expression value (black color).

The serum proteomic profile of the piglets fed C and CN containing diets such as T1 and T2 are presented in Figure 1. The DEPs in the serum of the piglets fed experimental diets were categorized according to the types of protein expressed individually (Figure 1A). Of the identified DEPs, a total of 103 DEPs were found to be significant, of which, 5, 7, and 10 DEPs were uniquely present in the serum of the piglets fed with the C, T1, and T2 diet groups, respectively, whereas 65 DEPs were commonly identified. Likewise, in comparison of the C and T1 diets, the piglets commonly shared the 8 DEPs in the serum and, 3 common DEPs for C and T2 as well as 3 DEPs for T1 and T2 diets (Figure 1A). In addition, we presented a hierarchical clustering heat map of DEPs identified from the proteomic analysis (Figure 1B). The results demonstrated the expression levels of DEPs largely associated with the blood serum of the weaned piglets fed T1 (0.5 mL CN/kg diet) and T2 (1.0 mL CN/kg diet) diets in comparison to the C (control) diet. 

Based on the log fold change in serum DEPs of piglets fed C, T1, and T2 diets, top 18 DEPs are presented in the Table 3. The results revealed that 14 DEPs were upregulated and 4 DEPs were downregulated. Among the upregulated proteins, Alpha-1-acid glycoprotein (ORM1) and Vitamin K-dependent protein S (PROS1) were found to be reduced/deregulated in piglets fed T1 diet; however, the proteins were highly upregulated in piglets fed T2 diet.

#### 3.3.2. Gene Ontology (GO) Terms of DEPs

**Figure 2:**
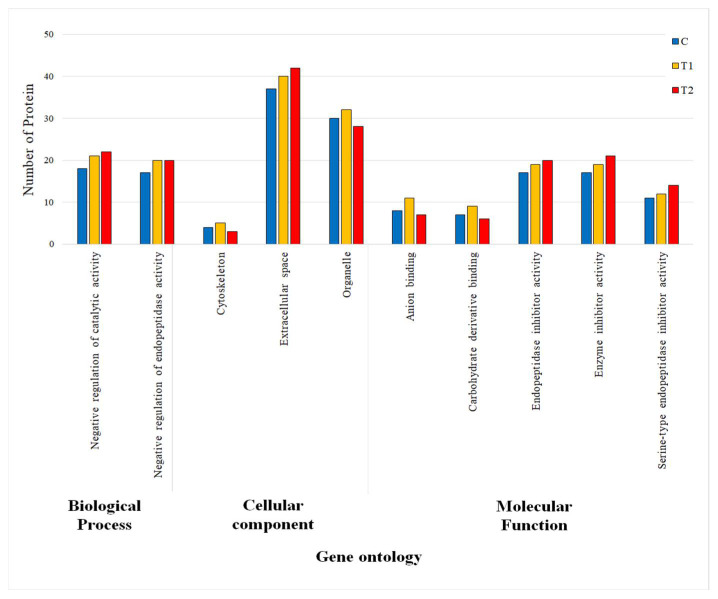
Gene ontology (GO) based on biological processes, cellular components and molecular functions of differentially expressed proteins (DEPs) in the piglets fed C, T1, and T2 diets.

For a better understanding, all data related to the GO terms based on biological processes, cellular components and molecular functions are presented in the Figure 2. In case of the biological processes of the piglets fed C and T1 diets, most of the protein functions are related to the negative regulation of catalytic activity and the negative regulation of endopeptidase activity. The piglets fed T1 and T2 diet showed a higher number of proteins functions for both of the activities of biological process compared to the C diet group (Figure 2). Considering the cellular component (Figure 2), a higher number of proteins found in the extracellular spaces, along with a moderate amount of proteins in cell organelles, but a little amount in the cell cytoskeleton in the piglets fed C, T1, and T2 diets. Based on the GO term analysis, all of the proteins for the cellular component were found to be greater in T1 than the piglets fed C diet except for the cell cytoskeleton and organelle in the piglets fed T2 diet (Figure 2). For the molecular function analysis of the DEPs in the piglets fed C, T1, and T2 diets (Figure 2), the results showed that the DEPs were exclusively associated with anion binding, carbohydrate derivative binding, endopeptidase inhibitor activity, enzyme inhibitor activity, and serine-type endopeptidase inhibitor activity, where the number of the proteins were slightly higher in the piglets fed T1 diet than in the piglets fed C diet.

#### 3.3.3. KEGG and REACTOME Pathway Analyses of DEPs

**Figure 3:**
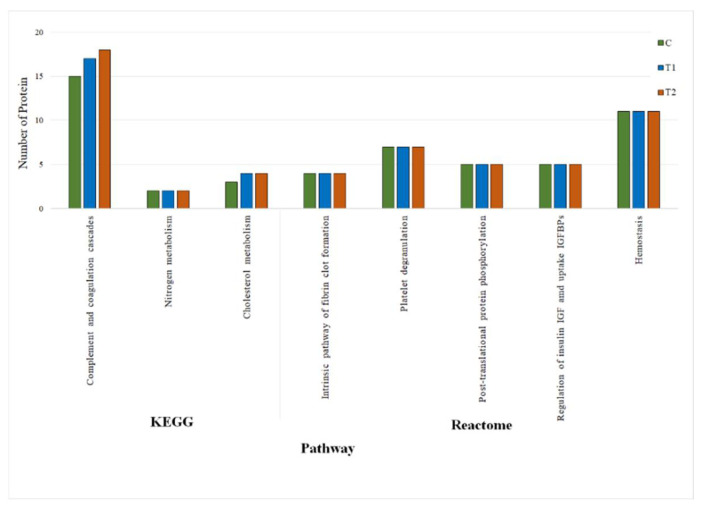
KEGG and REACTOME pathways classification and functional enrichment for the DEPs in the piglets fed C, T1, and T2 diets.

After KEGG and REACTOME pathways enrichment analyses for the DEPs in the piglets fed C, T1, and T2 diets (Figure 3), the results of the present study revealed that the KEGG enriched pathway mostly responded to complement and coagulation cascades, nitrogen metabolism, and cholesterol metabolism. The number of proteins associated with complement and coagulation cascades and cholesterol metabolism were found to be higher in piglets fed T1 and T2 diets than in the C diet. On the other hand, the REACTOME pathway associated with the fibrin clot formation, platelet degranulation, post-translational protein phosphorylation, regulation of insulin like growth factor (IGF) transport, and hemostasis. There were no differences in number of proteins related to the REACTOME pathways in piglets fed C, T1, and T2 diets.

#### 3.3.4. Protein–Protein Interaction (PPI) Network of DEPs

**Figure 4:**
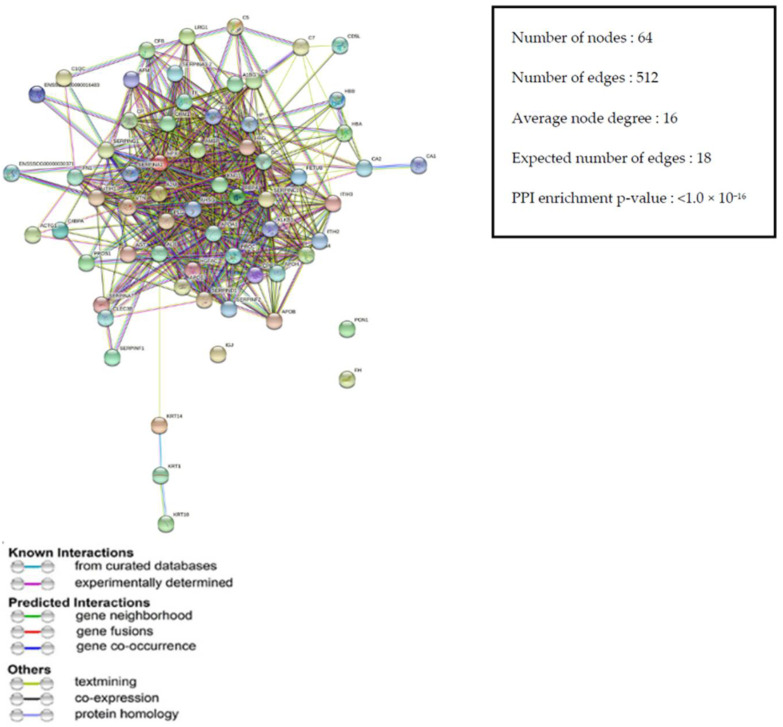
Protein–protein interaction (PPI) network for differentially expressed proteins (DEPs) analyzed by STRING software. The network nodes represent proteins (showing the interactions), and the round nodes denote individual proteins. The line color indicates the type of interaction evidence, and the line thickness indicates the strength of data support.

The PPI network analysis was constructed using the online STRING database for the differentially expressed proteins which were altered by the supplementation of CN in the diets of weaned piglets (Figure 4). The PPI network consisted of 64 nodes and 512 edges. The STRING analysis showed the presence of upregulated and downregulated proteins that mentioned in Table 3. Most of the connected proteins were associated with the stress response, regulation of catalytic activity, and immune response.

### 3.4. Fecal Coliform Bacteria

**Figure 5:**
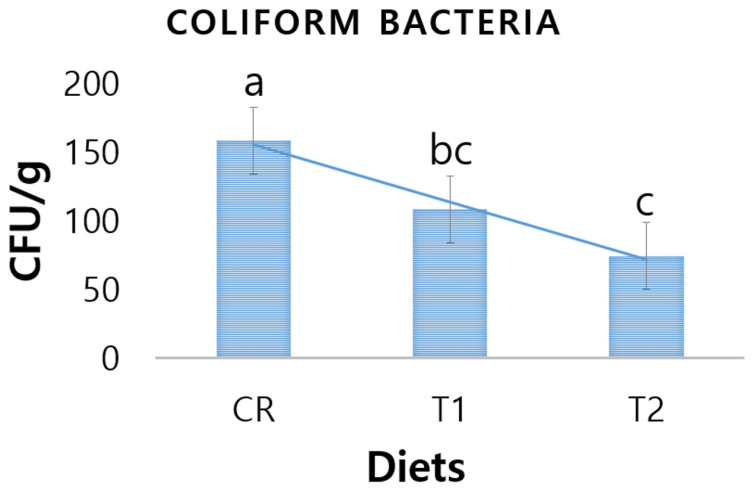
Number of coliform bacteria (CFU/g) in the feces of the weaned piglets fed control (CR), T1 (0.5 mL CN/kg diet) and T2 (1.0 mL CN/kg diet) diets. (a, b, c) are significantly different (*p* < 0.05) and the values in each row with same superscripts are non-significantly different.

Fecal coliform bacteria were significantly affected by dietary supplementation of CN in the diets of weaned piglets (Figure 5). At the end of the feeding trial, a significant level of reduction in the numbers of coliform bacterial colonies (CFU/g) was observed in the piglets fed T2 diet compared to the control diet (*p* < 0.05). However, there was no significant difference in fecal coliform bacteria count between the T1 and T2 diet groups (*p* > 0.05).

### 3.5. Fecal Ammonia and Hydrogen Peroxide

The effects of dietary CN in terms of fecal ammonia (NH_3_) and hydrogen sulfide (H_2_S) gas contents in the feces (25 g) of the weaned piglets are presented in the Table 4. The results showed that the piglets fed T1 and T2 diets had significantly lower fecal NH_3_ gas contents than those fed C diet. However, no significant difference in fecal NH_3_ malodors was observed between the T1 and T2 diet groups. Furthermore, all three groups exhibited a similar level of H_2_S gas contents in the feces of the weaned piglets.

## 4. Discussion

### 4.1. Growth Performance of Piglets

In the pharmacological perspective, the results revealed that the growth performance of the weaned piglets in terms of FW, WG, and ADG were improved with the supplementation of dietary CN at 0.5 mL (T1) or 1.0 mL (T2)/ kg diet. Moreover, significantly higher growth performance was observed in the T2 diet group compared with the control diet. In consistent of the present study, in a recent study, Marcon et al. [31] reported that dietary supplementation of curcumin-loaded nanocapsule (1.89 mg/kg) could significantly enhance the growth in terms of ADG and WG in lambs after a 17-day long experiment. Likewise, Reda et al. [45] reported the positive effects of nanocurcumin on the growth performance and feed utilization in terms of increased WG and decreased FCR in Japanese quails which is in agreement with the present study. However, Rahmani et al. [46] did not find any significant difference in the WG of broiler chicken fed high level of nanocurcumin (400 mg/kg diet) compared with the control diet under normal temperature. In case of feed utilization in weaned piglets, the results of the present study revealed that there were no significant differences in terms of FI and ADFI in the piglets fed C, T1, or T2 diets which may attributed to the well acceptance of the supplied experimental feeds and non-toxic effects of feeds by means of 100% survivability in piglets. Likewise, Marcon et al. [31] and Rahmani et al. [46] did not find any significant differences in the FI of lamb and broiler chicken, respectively, fed control and curcumin-loaded nanocapsule or nanocurcumin supplemented diets. In addition, Marchiori et al. [47] reported that the supplementation of nanocurcumin in the diet of quails can enhance the growth and egg quality of quails in cold stress situation. Furthermore, Lei and Kim [42] found that zinc oxide coated with lipid particle can significantly improve the growth and feed efficiency in young pigs which is in agreement with the present study for nanocurcumin coated with phospholipid in weaned piglets.

### 4.2. Serum Biochemistry of Piglets

Blood serological information can serve as an important tool to ascertain the first line of immunity in living animals. In the present study, serum GLU, GGT, AMYL, and LYPS contents did not significantly vary in the piglets of the control, T1, and T2 diet groups. This can be explained by the possibility that the doses of CN were non-hazardous to the health status and did not affect the digestibility in terms of carbohydrate (amylase, AMYL enzyme activity) and lipid (lipase, LYPS enzyme activity) metabolism. In consistency with our study, Marcon et al. [31] reported that the young lambs fed 1 mg/kg diet of curcumin-loaded nanocapsule did not show any significant changes in serum GLU and GGT levels compared to the control diet. Serum TBIL, ALT, AST, and ALKP are the indicators of liver function where higher levels of these parameters indicate deleterious conditions of the body. In this study, serum TBIL level was not significantly affected by the T1 diet, however, a higher dose of CN (T2 diet, 1.0 mL/kg) significantly increased the serum ALT and ALKP. According to the ALT and ALKP data in our study, it seemed that higher levels of ALT and ALKP were not toxic to the liver, as reflected by the increment of growth with a significantly higher weight gain in the T2 group compared to the control diet. In consistency with the present study, Marcon et al. [31] found a significantly higher level of AST in lambs fed 2 mg/kg diet of curcumin-loaded nanocapsule compared with the control diet. Likewise, Reda et al. [45] reported that dietary nanocurcumin at 0.5 g/kg of diet could significantly increase the ALT level in Japanese quails as a monogastric animal model, in agreement with the present study. Serum ALB protein helps to balance the blood osmotic pressure between the blood vessels and tissues; and GLB is produced in the liver, which is responsible for proper function of liver, clotting of blood, and fighting against infections. In the present study, serum total protein showed a significantly greater amount in the piglets of the high CN diet, in agreement with Reda et al. [45]. Serum ALB levels were significantly higher in the piglets fed T1 and T2 diets compared to the control diet; however, serum GLB level as well as ALB and GLB ratio were not affected by the dietary treatments. On the contrary, Marcon et al. [31] found no significant difference in serum ALB content in the lambs fed 0, 1, 2, 3, or 4 mg/kg diet of curcumin-loaded nanocapsule; and serum GLB contents were higher in the lambs fed higher levels of curcumin-loaded nanocapsule. Furthermore, Reda et al. [45] reported significant increment of ALB and GLB levels in the Japanese quails fed higher levels of nanocurcumin in the diets. Serum CRT and BUN are commonly associated with the functions of kidney in animals. Unusually high levels of CRT and BUN in blood serum may indicate the improper functioning of the kidneys. In the present study, the piglets fed higher level of CN showed significantly higher levels of serum CRT and BUN levels. However, CRT and BUN ratio did not show any significant change among the treatment groups, suggesting that the serum CRT and BUN levels were not toxic to the kidney tissues of the animals. In addition, in this study, serum CRT (1.00–2.60 mg/dL) and BUN (8.00–24.0 mg/dL) levels were found to be in suitable ranges for normal pigs based on Mitruka and Rawnsley [48]. In agreement with our study, Marcon et al. [31] found a significantly increased urea contents in lambs supplemented with higher level of nanocurcumin in the diets. However, Reda et al. [45] observed no significant changes in serum CRT and urea contents in the Japanese quails fed nanocurcumin in the diet. Serum electrolytes such as sodium, calcium, chloride, potassium, and phosphorus are known to contribute to maintaining body fluids in living animals. In the current study, serum IP and Ca were found to be significantly higher in the T2 diet which might helped to achieve the highest growth performance and health status among the piglets. Interestingly, TCHOL was significantly reduced in the piglets fed CN supplemented diets. In line with the present study, Reda et al. [45] also reported that dietary supplementation with nanocurcumin could significantly reduce the total cholesterol levels in the blood serum and the liver of the weaned piglets with intrauterine growth retarded (IUGR). Overall, in the present study, the serum biochemical parameters in weaned piglets fed C, T1, and T2 diets were found to be normal [48].

### 4.3. Serum Proteomics of Piglets

The proteomic profile based on bioinformatics tools in a pre-clinical study on animal model can provide important attributes on the efficacy of biomedicine in terms of physiology, toxicity, and immunity of the animal on molecular level [43]. In the present study, we conducted a comparison based on a proteomic analysis of the blood serum, among the experimental piglet groups through the dietary administration of different CN levels as opposed to the control diet. We also interpreted the bioinformatics data based on the gene ontology (GO) terms regarding biological processes, cellular components, and molecular functions as well as the pathway enrichment analyses in terms of KEGG and REACTOME pathway associated with the pig, *Sus scrofa*. The results of the present study indicate that 103 DEPs were highly expressed in serum of the piglets fed C, T1, and T2 diets, of which, 65 DEPs were commonly identified in piglets fed the diets. Likewise, Liu et al. [37] reported 64 DEPs in the piglets fed garlic botanical. In this study, among the 65 DEPs, 18 DEPs were selected where 14 DEPs (APOA4, SERPINC1, PROC, C6, ADIPOQ, CRP, JCHAIN, C1QC, KRT14, THBS1, C9, VTN, ORM1, PROS1) were found to be upregulated and 4 DEPs (FN1, C5, KRT10, CA2) were downregulated based on log fold change of ≥1 (upregulated) or ≤−1 (downregulated), in the piglets fed T1 and T2 diets compared to the C diet, respectively. Furthermore, among the 14 upregulated DEPs, there were 7 DEPs such as APOA4, PROC, C6, C1QC, KRT14, VTN, and PROS1 found to be highly upregulated in the blood serum of the weaned piglets fed T2 diet compared to the T1 diet which attributed to the enhancement of health status of pigs.

In this study, based on the biological process of GO terms, most of the proteins involved in negative regulation of endopeptidase activity (GO:0010951) and negative regulation of catalytic activity (GO:0043086) in piglets fed C, T1 and T2 diets; those proteins were highly expressed in the piglets fed CN supplemented diets compared to the control diet. Regarding cellular components, most of the DE proteins were highly associated with the extracellular space (GO:0005615) outside the plasma membrane occupied by the cell fluid and organelles (GO:0043226) such as the nucleus, mitochondria, vacuoles, vesicles, ribosomes as well as cell cytoskeletons (GO:0005856). Furthermore, the highest amount of protein was found in extracellular spaces in serum of the piglets fed C, T1, and T2 diets. For molecular function, five sets of DEPs were highly expressed for enzyme inhibitor activity (GO:0004857) binding to and stop, prevent or reduces the activity of an enzyme; for anion binding (GO:0043168) interacting with charged atoms or groups of atoms with a net negative charge; for endopeptidase inhibitor activity (GO:0004866) binds to and stop, prevents or reduces the activities of endopeptidase enzymes; for carbohydrate derivative binding (GO:0097367) that interacts selectively or non-covalently with carbohydrate derivatives; and for serine-type endopeptidase inhibitor activity (GO:0004867) binding to and stops, prevents, or reduces the activity of serine-type endopeptidase enzymes.

In the case of the KEGG pathway enrichment, we identified three significantly enriched pathways such as complement and coagulation cascades, nitrogen metabolism, and cholesterol metabolism. The complement systems are the main parts of innate immune system and coagulation cascades related to blood coagulation or hemostasis [49]. In this study, the complement systems (C6 and C9), and blood coagulating proteins like antithrombin-III (SERPINC1) and vitamin-K-dependent protein C (PROC) were highly upregulated in piglets fed CN supplemented diets which ultimately increased the health status of piglets in terms of growth performance. Moreover, as the nitrogen metabolism related protein like carbonic anhydrase 2 (CA2) was downregulated and cholesterol metabolism related protein like apolipoprotein A-IV (APOA4) was upregulated, we assumed that these proteins were responsible for reducing ammonia gas contents in feces and cholesterol levels in serum of the piglets fed CN supplemented diets that have been evidenced in the fecal malodors and serum biochemical assays in the present study. The relation between carbonic anhydrase and ammonia gas is also reported in other findings [50,51]. The REACTOME pathway revealed the five pathways were involved in protein enrichment such as intrinsic pathway of fibrin clot formation, platelet degranulation, post-translational protein phosphorylation related to protein synthesis, regulation of insulin like growth factor (IGF) transport, and uptake related to extracellular region and hemostasis related to systemic blood regulations. Based on the KEGG and REACTOME pathway analyses, we found that majority of the proteins were involved to enhance immune status, high response to drug and metabolism in the piglets fed CN supplemented diets.

The online STRING network revealed that the upregulated and downregulated proteins were mostly related to the stress response, regulation of catalytic activity, and immune response in terms of upregulating the blood coagulation (SERPINC1, PROC); innate and adaptive immune response (C6, CRP, JCHAIN, C1QC); skeletal protein (KRT14); cellular response to drug (ADIPOQ), lipid metabolism (APOA4), inhibition of cell membrane damage (VTN); and downregulating the fibrin clot formation (FN1), fibrous protein (KRT10), and intracellular pH in intestine. Overall, the STRING network showed the positive effect of CN supplementation as a biomedicine in the diet of weaned piglets.

### 4.4. Fecal Pathogenic Bacteria of Piglets

In the weaning period of piglets, diarrhea is the most common medical condition that arises from the significant changes in their gastrointestinal tract (GIT) physiology, microbiology, as well as immunology [48,52]. In this study, fecal coliform bacteria were found to be significantly reduced by the dietary supplementation with CN in the weaned piglets. In line with our study, Lei and Kim [42] reported that dietary lipid-coated zinc nanoparticles showed a positive effect on reducing fecal coliform bacteria in young pigs challenged with enterotoxigenic *E. coli* bacteria. Likewise, Rahmani et al. [46] postulated that dietary nanocurcumin can effectively reduce the cecal *E. coli* populations.

### 4.5. Fecal Malodors of Piglets

The odor emissions from swine farm operations are a major concern worldwide which extremely affects the air quality, quality of human life and health status, and the property values of the houses in the close proximities of the swine farm facilities [53]. In the present study, we found that CN supplemented diets can reduce the fecal malodors such as NH_3_ gas of the piglets, thus constituting a great significance in environment friendly rearing of pigs in the farm level. Similarly, in our previous research on CN effect on mice, we reported that positive effects of the dietary CN on reducing fecal NH_3_ gas [54].

## 5. Conclusions

In conclusion—based on the growth, feed utilization, serum biochemistry and proteomics, fecal coliform bacteria and noxious gas like NH_3_—the dietary supplementation of CN can be recommended between 0.5 mL/kg and 1.0 mL/kg as an alternative of antibiotics for weaned young pigs. The results of the present study demonstrated the successful application of nanocurcumin with lipid carriers capable of increasing the growth and production of pigs as well as improving the air environment by reducing malodors. Further studies are warranted for the application of CN in other animal models in the field of nanobiotechnology.

## Figures and Tables

**Table 1:** Effects of dietary curcumin nanospheres (CN) on growth performance and feed utilization in weaned piglets for 21-days ^1^.

Items	Dietary Treatments			*p*-Value
	Control, C	T1 (0.5 mL CN/kg)	T2 (1.0 mL CN/kg)	
FW ^2^	13.60 ± 0.04 ^b^	14.66 ± 0.76 ^ab^	15.44 ± 0.35 ^a^	0.0098
WG ^3^	5.98 ± 0.55 ^b^	6.92 ± 0.46 ^ab^	7.68 ± 0.59 ^a^	0.0228
ADG ^4^	0.285 ± 0.03 ^b^	0.329 ± 0.02 ^ab^	0.366 ± 0.03 ^a^	0.0223
FI ^5^	9.76 ± 0.06 ^a^	10.07 ± 0.45 ^a^	10.65 ± 0.51 ^a^	0.0778
ADFI ^6^	0.465 ± 0.00 ^a^	0.479 ± 0.02 ^a^	0.507 ± 0.02 ^a^	0.0787
FE ^7^	61.28 ± 5.53 ^b^	68.65 ± 1.60 ^ab^	72.08 ± 2.18 ^a^	0.0251
FCR ^8^	1.64 ± 0.14 ^a^	1.46 ± 0.03 ^ab^	1.38 ± 0.04 ^b^	0.0280

^1^ Values are mean ± SD from triplicate groups of piglets (*n* = 3) where values in each row with different superscripts are significantly different (*p* < 0.05) and the values in each row with same superscripts are non-significantly different. ^2^ Final weight (kg/piglet). ^3^ Weight gain (kg/piglet) = (final weight − initial weight)/initial weight. ^4^ Average daily gain. ^5^ Feed intake (kg/piglet). ^6^ Average daily feed intake; ^7^ Feed efficiency (%/pig) = weight gain × 100/feed intake. ^8^ Feed conversion ratio = feed intake/weight gain. Values in each row with different superscripts ^a, b^ are significantly different (*p* < 0.05) and the values in each row with same superscripts are non-significantly different.

**Table 2:** Effects of dietary curcumin nanospheres (CN) on serum biochemistry of weaned piglets for 21-days ^1^.

Items	Dietary Treatments			*p*-Value
	Control, C	T1 (0.5 mL CN/kg)	T2 (1.0 mL CN/kg)	
GLU	107.3 ± 0.6 ^a^	106.7 ± 0.6 ^a^	106.7 ± 0.6 ^a^	0.331
CRT	1.2± 0.1 ^b^	1.3 ± 0.1 ^b^	1.5± 0.1 ^a^	0.002
BUN	7.7± 0.6 ^b^	8.3 ± 0.6 ^ab^	9.3 ± 0.6 ^a^	0.033
BUN:CRT	6.4 ± 0.3 ^a^	6.2 ± 0.1 ^a^	6.2 ± 0.3 ^a^	0.448
IP	8.7 ± 0.1 ^c^	9.2 ± 0.1 ^b^	9.8 ± 0.1 ^a^	0.001
Ca	10.3 ± 0.1 ^c^	11.2 ± 0.1 ^b^	11.5 ± 0.2 ^a^	0.001
T-Pro	6.8 ± 0.1 ^b^	6.8 ± 0.1 ^b^	7.0 ± 0.1 ^a^	0.001
ALB	2.8 ± 0.1 ^c^	3.3 ± 0.2 ^b^	4.2 ± 0.1 ^a^	0.001
GLB	4.1 ± 0.1 ^a^	4.1 ± 0.1 ^a^	4.0 ± 0.1 ^a^	0.317
ALB:GLB	0.7 ± 0.1 ^a^	0.8 ± 0.1 ^a^	0.9 ± 0.1 ^a^	0.072
ALT	27.0 ± 1.0 ^b^	30.0 ± 2.0 ^ab^	33.0 ± 2.0 ^a^	0.015
ALKP	85.0 ± 1.0 ^b^	86.3 ± 1.0 ^b^	91.0 ± 1.0 ^a^	0.010
GGT	8.0 ± 1.0 ^a^	8.0 ± 1.0 ^a^	8.0 ± 1.0 ^a^	1.000
TBIL	0.4 ± 0.1 ^a^	0.3 ± 0.1 ^a^	0.4 ± 0.1 ^a^	0.422
TCHOL	145.7 ± 2.1 ^a^	133.7 ± 1.5 ^b^	124.7 ± 1.2 ^c^	0.001
AMYL	126.3 ± 1.5 ^a^	123.0 ± 1.0 ^a^	125.7 ± 1.5 ^a^	0.054
LYPS	1339.3 ± 17.8 ^a^	1335.3 ± 18.1 ^a^	1358.0 ± 7.0 ^a^	0.230

^1^ Values are mean ± SD from triplicate groups of piglets (*n* = 3) where values in each row with different superscripts ^a, b, c^ are significantly different (*p* < 0.05) and the values in each row with same superscripts are non-significantly different. Glu, glucose (mg/dL); CRT, creatinine (mg/dL); BUN, blood urea nitrogen (mg/dL); IP, inorganic phosphorus (mg/dL); Ca, calcium (mg/dL); T-Pro, total protein (g/dL); ALB, albumin (g/dL); GLB, globulin (g/dL); ALT, alanine aminotransferase; ALKP, alkaline phosphatase; GGT, gamma-glutamyl transferase; TBIL, total bilirubin; TCHOL, total cholesterol; AMYL, amylase; LYPS, lipase.

**Table 3:** List of differentially expressed proteins (DEPs) identified (Top 18) as upregulated (≥1) and downregulated (≤−1) proteins based on log2 fold change, log2 (FC) data in piglets fed T1 and T2 diets in comparison to the control diet.

Regulation	Acsession Number	Protein Name	Gene Symbol	Log2 (FC) ^1^		Main Function
				T1	T2	
Up	F1SJT7	Apolipoprotein A-IV	APOA4	0.92	1.96	Lipid metabolism
	F2Z5E2	Antithrombin-III	SERPINC1	7.93	7.84	Regulate blood coagulation
	Q9GLP2	Vitamin K-dependent protein C	PROC	3.17	5.35	Regulate blood coagulation
	F1SMI8	Complement C6	C6	1.00	3.45	Innate and adaptive immune response
	A0A4X1U519	C1q domain-containing protein	ADIPOQ	3.80	4.39	Cellular response to drug
	O19062	Pentaxin	CRP	2.58	3.00	Innate immune response
	F1RUQ0	Immunoglobulin J chain	JCHAIN	3.00	3.00	Secretion of IgA and IgM into mucosa
	A0A286ZSJ7	Complement C1q subcomponent subunit C	C1QC	1.00	4.00	Innate immune system
	A0A287AXN9	IF rod domain-containing protein	KRT14	1.00	4.08	Fibrous protein in cells of skin, hair, nail
	F1SS26	Thrombospondin-1	THBS1	4.00	1.00	Mediates cell-to-cell and cell-to-matrix interactions
	F1SMJ6	Complement component C9	C9	0.28	1.44	Innate immune system
	P48819	Vitronectin	VTN	0.74	1.29	Inhibit cell membrane damage
	F1SN68	Alpha-1-acid glycoprotein	ORM1	−0.28	1.06	Transport protein in blood stream
	F1SK70	Vitamin K-dependent protein S	PROS1	−0.58	2.50	Prevent coagulation and stimulate fibrinolysis
Down	F1SS24	Fibronectin	FN1	−0.14	−1.93	Blood coagulation, fibrin cloat formation
	Q6VPV1	Complement C5a anaphylatoxin	C5	−0.54	−1.36	Smooth muscle contraction, basophil and mast cell degranulation
	I3LDS3	Keratin 10	KRT10	−1.13	−1.23	Fibrous protein in cells of skin, hair, nail
	F1RXC2	Carbonic anhydrase 2 isoform 1	CA2	0.74	−4.95	Intracellular pH regulation in intestine

^1^ FC = fold change; log2 (FC) of T1 = T1/control; log2 (FC) of T2 = T2/control.

**Table 4:** Effects of dietary curcumin nanospheres (CN) on ammonia (NH_3_) and hydrogen sulfide (H_2_S) gas contents in feces of weaned piglets for 21-days ^1^.

Items	Dietary Treatments			*p*-Value
	Control, C	T1 (0.5 mL CN/kg)	T2 (1.0 mL CN/kg)	
Ammonia (ppm)	6.33 ± 0.58 ^a^	1.33 ± 0.58 ^b^	1.67 ± 0.58 ^b^	0.0001
Hydrogen sulfide (ppm)	0.27 ± 0.06 ^a^	0.17 ± 0.06 ^a^	0.27 ± 0.06 ^a^	0.1250

^1^ Values are mean ± SD from triplicate groups of piglets (*n* = 3) where values in each row with different superscripts are significantly different (*p* < 0.05). Values in each row with different superscripts ^a, b^ are significantly different (*p* < 0.05) and the values in each row with same superscripts are non-significantly different.

## Data Availability

All data reported in this article.
